# Accuracy of Electrode Placement in IRE Treatment with Navigated Guidance

**DOI:** 10.1007/s00270-020-02762-5

**Published:** 2021-01-20

**Authors:** David Stillström, Raluca-Maria Sandu, Jacob Freedman

**Affiliations:** 1 Division of Surgery, Department of Clinical Sciences, Karolinska Institutet at Danderyd Hospital, Stockholm, Sweden; 2 ARTORG Centre for Biomedical Engineering Research, University of Bern, Bern, Switzerland; 3 Department of Surgery and Urology, Danderyd Hospital, 182 88 Stockholm, Sweden

**Keywords:** Liver, Ablation, IRE, Navigation, Stereotactic, Hepatocellular carcinoma

## Abstract

**Purpose:**

Evaluate the accuracy of multiple electrode placements in IRE treatment of liver tumours using a stereotactic CT-based navigation system.

**Method:**

Analysing data from all IRE treatments of liver tumours at one institution until 31 December 2018. Comparing planned with validated electrode placement. Analysing lateral and angular errors and parallelism between electrode pairs

**Results:**

Eighty-four tumours were treated in 60 patients. Forty-six per cent were hepatocellular carcinoma, and 36% were colorectal liver metastases. The tumours were located in all segments of the liver. Data were complete from 51 treatments. Two hundred and six electrodes and 336 electrode pairs were analysed. The median lateral and angular error, comparing planned and validated electrode placement, was 3.6 mm (range 0.2–13.6 mm) and 3.1° (range 0°–16.1°). All electrodes with a lateral error >10 mm were either re-positioned or excluded before treatment. The median angle between the electrode pairs was 3.8° (range 0.3°–17.2°). There were no electrode placement-related complications.

**Conclusion:**

The use of a stereotactic CT-based system for navigation of electrode placement in IRE treatment of liver tumours is safe, accurate and user friendly.

## Introduction

Local tumour ablation is well established in the treatment of liver tumours, both primary and metastatic cancers [1, 2]. The most commonly used methods are radio frequency ablation (RFA) and microwave ablation (MWA) [3, 4]. Both these methods are thermal and use heat to create coagulative necrosis of the tumour. One limitation of these methods is the inability to treat tumours close to major hepatic vessels, bile ducts and other heat-sensitive structures. This is where irreversible electroporation (IRE) has its primary role in treatment of hepatic malignancies [1, 3].

IRE is performed by applying short pulses with high voltage direct current across the tumour. The electrical pulses are delivered pairwise through two to six electrodes placed around the tumour. This leads to the formation of nano-pores through the cell membranes, disrupting the cells’ homeostasis causing them to go into apoptosis [1, 5–7]. Since this effect is created without any significant temperature rise in the periphery, it is possible to treat tumours close to heat sensitive structures.

The two major problems with ablative treatments in general are predictability and precision in delivering the modality chosen. Precision is mainly concerned with the accurate placement of applicators. This is usually accomplished with navigation under radiological guidance, either percutaneously or during laparoscopic or open surgery. The applicator can be guided to the desired location with ultrasound, ultrasound with fused computed tomography (CT) images or magnetic resonance images (MRI), conventional CT or stereotactic CT-guidance.

The safety and efficacy of stereotactic CT-guidance for placement of single applicators in RFA and MWA treatments has been proven in several studies [8–11].

Multiple placements of electrodes in IRE treatment are more demanding. The tumours are often in the central part of the liver, close to larger bile ducts and major hepatic vessels. The demand of parallelism also makes the electrode placement more demanding compared to the placement of a single applicator. The electrodes are placed around the tumour about 20 mm apart, covering a larger volume, compared to a single applicator where the aim is to place it in the centre of the tumour. In ultrasound guided IRE, the electrode placement can be challenging since the point where the electrode is aimed is off target, i.e. outside the tumour. Different methods of computer assisted guidance have been developed [12]. There are publications showing initial experiences with stereotactic CT navigation and robot assisted navigation with good results compared to conventional CT guided placement [13, 14].

The aim of this retrospective study is to evaluate the accuracy of multiple electrode placements in IRE treatment of liver tumours with guidance using a stereotactic CT-based system in clinical practice.

## Materials and Methods

### Patient Selection

All patients were referred for ablative treatment after discussion on a liver specific multidisciplinary team conference (MDT). IRE treatment was chosen for tumours that were neither suitable for resection, for various reasons, nor thermal ablation due to proximity to heat sensitive structures, i.e. central bile ducts or other organs where organ displacement with hydro dissection or laparoscopy was deemed inappropriate.

### Procedure

The procedures were performed under general anaesthesia with full muscle relaxation. To minimize movement of the liver during respiration, high-frequency jet ventilation (HFJV) was used [15–18].

The procedures were performed percutaneously in the radiology department. The electrodes were placed with guidance of the CAS-ONE system (CAScination AG, Bern, Switzerland).

**Fig. 1:**
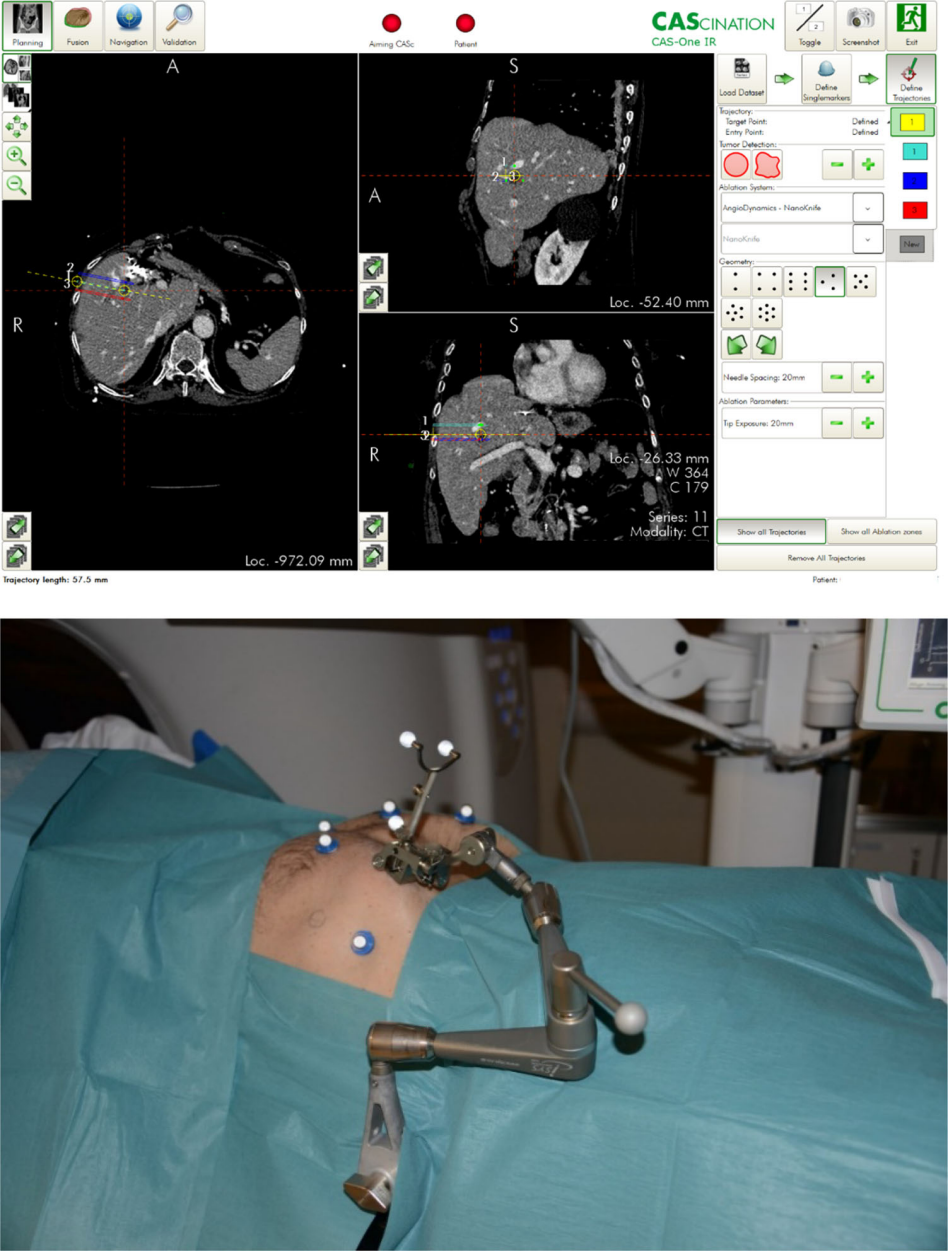
View of CAS-ONE work screen (top) and adjustable arm (bottom)

CAS-ONE is an optical stereotactic CT-guided navigation system that can be used for placement of applicators during ablative therapies. The system consists of a computer with two touch screens, two infrared cameras, and a seven-degree of freedom adjustable arm with an electrode guide for fixing the applicator insertion point and angle. The patient is placed on a carbon fibre plate on the CT table, and six retro-reflective skin markers are glued to the skin in a pre-designed pattern. A diagnostic CT-scan with intravenous contrast enhancement is performed and uploaded into the system. The skin markers, bone structures and skin are automatically segmented by the system, creating a 3D view of the patient on the screens. The CT images can be simultaneously visualized in six different projections. Firstly, the centre of the tumour and the desired skin entry area of the electrodes are marked on the screen, denoted as the reference trajectory. Secondly, the system allows choosing the desired electrode configuration pattern for 2–7 electrodes. The entire group of electrodes can then be adjusted together to keep them parallel when rotating or changing the angles to avoid bone structures, blood vessels and bile ducts. Every electrode can also be adjusted individually. Thirdly, the adjustable arm is set in position for each electrode and locked. The electrode is then inserted through the applicator guide at the tip of the aiming device (see Fig. 1). Description of the system has previously been published [9, 11, 19].

When all electrodes are in place, a CT scan without contrast is performed and uploaded into the CAS-ONE. The CT series are co-registered automatically by the CAS-ONE system using rigid registration. To confirm that the liver has not moved and that the electrodes are in the correct position around the tumour, the two CT scans are overlaid with a transparency gradient using the computer-assisted system. The system can also detect the electrodes and compare the planned trajectory with the actual placement, and the distances between the electrodes are provided by the system. In a previous software version, this had to be done manually.

The treatments were performed with NanoKnife® (Angiodynamics; Latham, NY, USA) according to the manufacturer’s instructions. 10–20 test pulses were delivered between each pair of electrodes. The delivered current was analysed and adjusted if needed before a minimum of 70 treatment pulses were delivered. The delivered current was again analysed regarding change in delivered amperage according to the manufacturer’s recommendations, and further treatment was delivered if necessary.

Last, after completion of the pulse deliveries, a CT scan was performed with the patient still under general anaesthesia, using intravenous contrast enhancement, kidney function allowing, to evaluate immediate ablation result and early complications.

### Data Collection

**Fig. 2:**
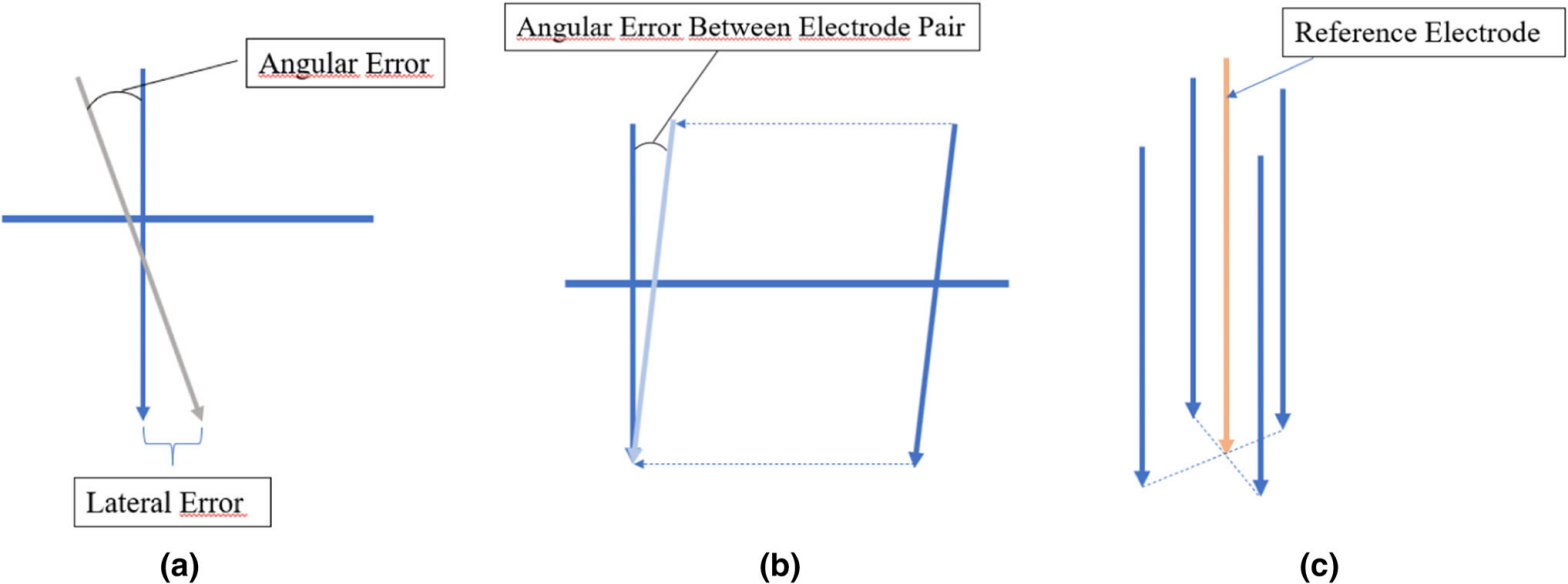
**a** Angular and lateral error between planned and validated electrode placement **b** Angular error between two validated electrodes in a pair **c** Relationship between the centre reference electrode and four validated electrodes

A retrospective analysis of all CT images was performed. All planned electrode placements were compared with actual placements. The lateral and angular error for each electrode was calculated (Fig. 2a). The angular error between each electrode pair was calculated as a measurement of parallelism, see Fig. 2b. The angular error between each electrode and the reference trajectory was also measured, see Fig. 2c.

### Statistical Analyses

Descriptive statistics were used for presentation of patient characteristics.

STATA 15 (StataCorp, College Station, Texas 77845 USA) was used for the statistical analyses.

### Ethical Approval

Ethical approval was obtained from the regional ethical review board in the Stockholm–Gotland region (EPN Dnr2016/2212-31/2). Patient informed consent for this study was waived because of its retrospective nature.

## Results

### Patients

**Fig. 3:**
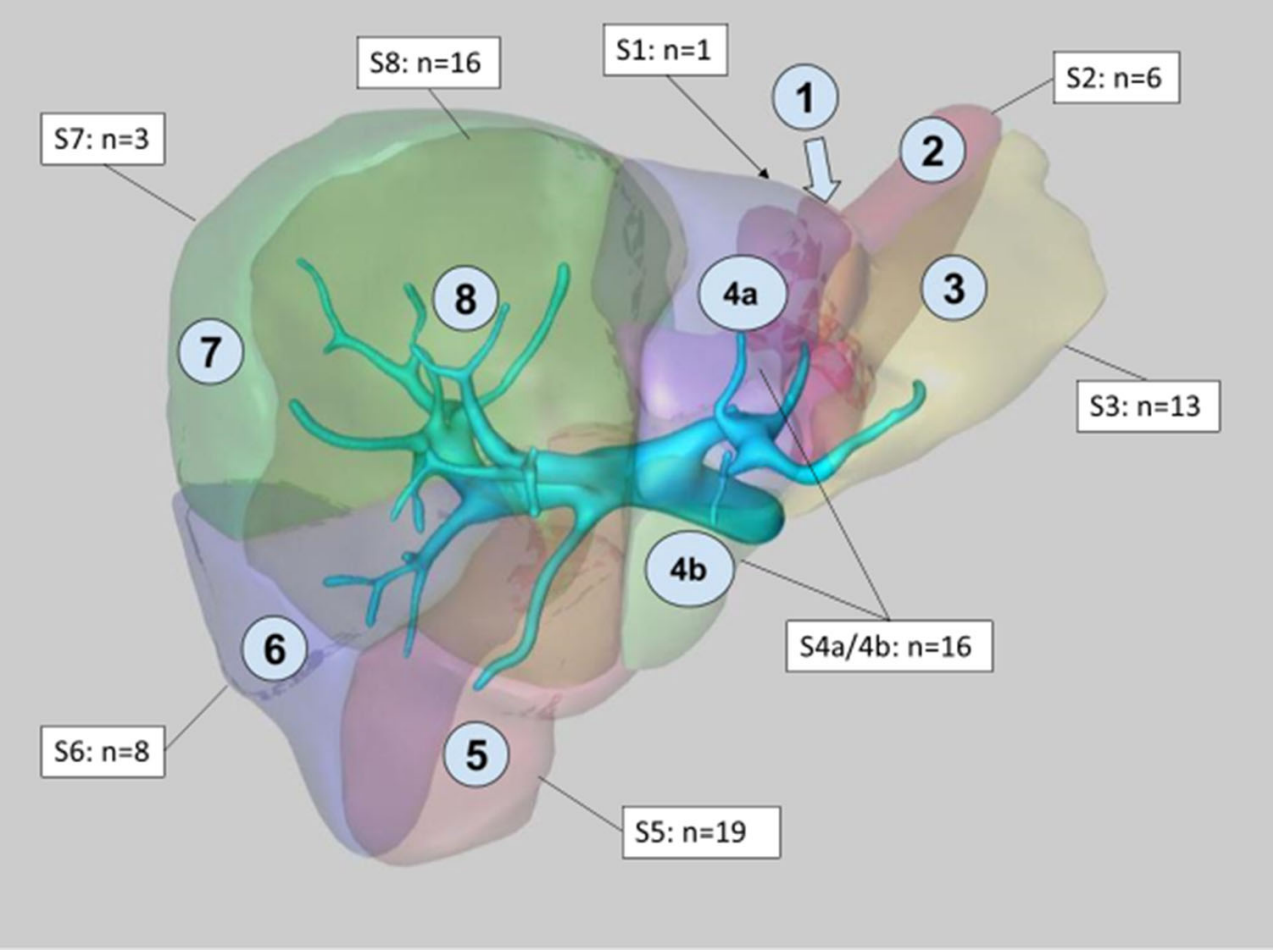
Distribution of tumours in the liver

Sixty patients with 84 tumours were treated. The most common diagnoses were hepatocellular carcinoma (HCC) 43% and colorectal cancer liver metastases (CRCLM) 36%. Mean tumour diameter was 21 mm, the median number of electrodes used was 4, and the total number of electrodes used were 300. See Table 1. The tumours were located in all segments of the liver, see Fig. 3.

**Table 1:** Patient and tumour characteristics

*Sex, no. of patients (%)*	
Male	58 (81%)
Female	14 (19%)
Age (y), mean (±SD)	65.0 ± 11.0
*Tumour type, no (%) of tumours*	
Colorectal liver metastases	30 (35.7%)
Hepatocellular carcinoma	36 (42.9%)
Cholangiocarcinoma	4 (4.8%)
Livermetastes from CCC	2 (2.4%)
Leiomyosarcoma	1 (1.2%)
Sarcoma	1 (1.2%)
Adrenocortical carcinoma	7 (8.3%)
Pankreas NET	2 (2.4%)
Tumour diameter (mm), median (min-max)	19 (2–60)
Number of electrodes/tumour, median (min-max)	4 (2–6)
Number of electrodes, total	300
DPL, Patient radiation dose (mGy x cm), mean (±SD)	1654.9 ± 686.0

### Electrode Accuracy

In the analyses of electrode placements, data were missing for 12 treatments and these patients were hence excluded from the analyses. Furthermore, five patients were excluded since the registration was inaccurate due to large movements of the liver during the procedure. In the analyses of angular error between electrode and reference trajectory, the first version of the software did not give the interventionist the possibility to mark this, and therefore, the first eight treatments were not included in this part of the analysis.

In total, 51 treatments with 206 electrodes and 336 electrode pairs were analysed.

**Fig. 4:**
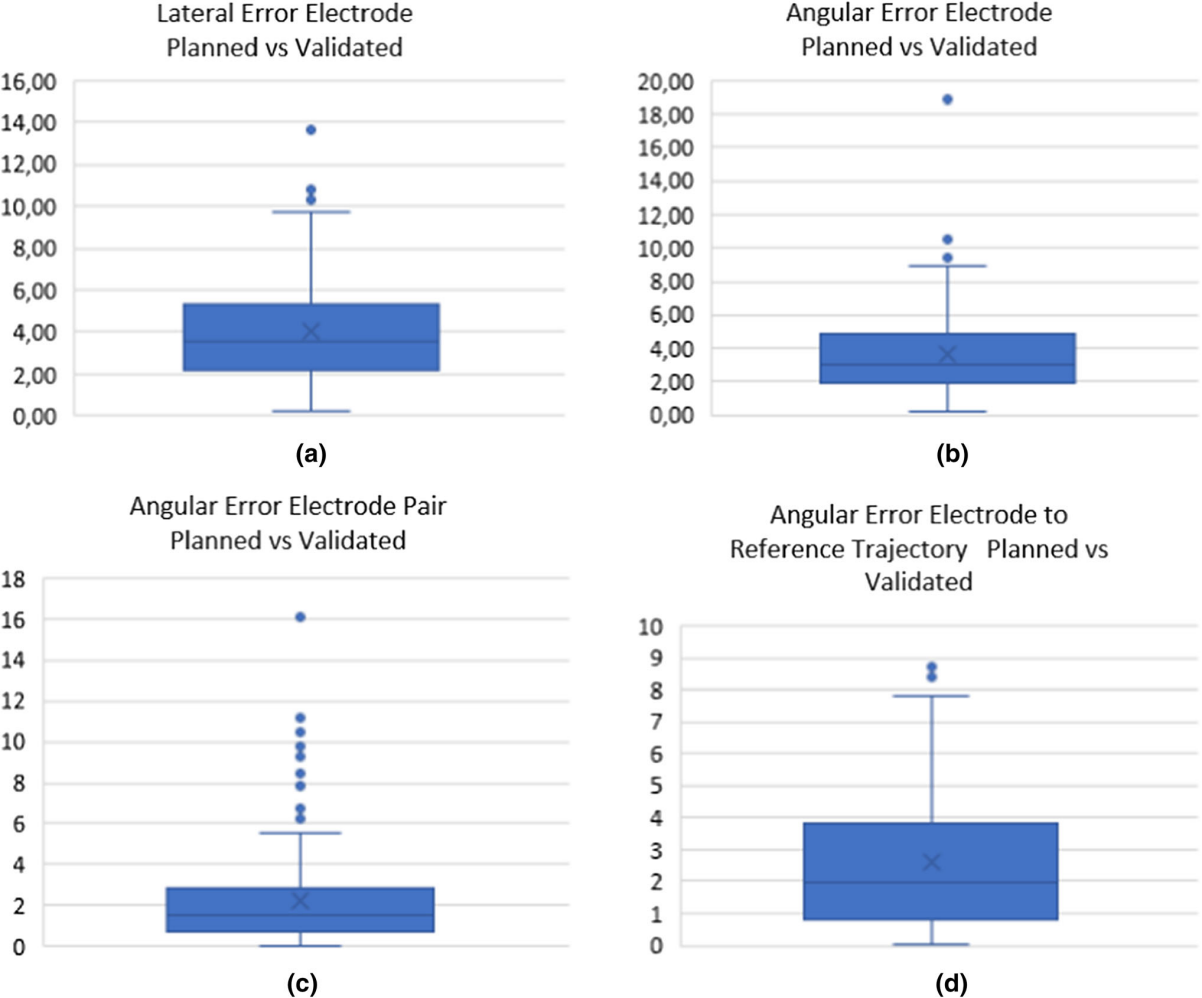
**a** Lateral error in mm between planned and validated electrode placement **b** Angular error in degrees between planned and validated electrode placement **c** Angular error in degrees between planned and validated electrode pairs **d** Angular error in degrees, comparing planned angel between electrode and reference electrode with validated angel between electrode and reference electrode

The median lateral and angular error, comparing planned and validated electrode placement, was 3.59 (range 0.2–13.64) mm and 3.06 (range 0.18–18.91) degrees. The median angular error for each electrode pair was 1.54 (range 0–16.11) degrees. The results for lateral and angular errors comparing planned and validated electrode placement are presented in Table 2 and Fig. 4.

**Table 2:** Lateral and angular errors

*Lateral error per electrode planned vs validated*	
Median	4.05
Range	0.2–13.64
*Angular error per electrode planned vs validated*	
Median	3.62
Range	0.18–18.91
*Angular error electrode pair*	
Median	2.25
Range	0–16.11
*Angular error electrode vs reference trajectory*	
Median	2.48
Range	0.01–8.68

All electrode placements were validated with a CT scan before treatment, and all with a lateral error >10 mm was either re-positioned before treatment or not used during the treatment due to sub-optimal placement. None of these patients suffered from any kind of electrode placement-related complications, i.e. bleeding, bile leakage or bowel injury.

### Electrode Pair Parallelism

**Fig. 5:**
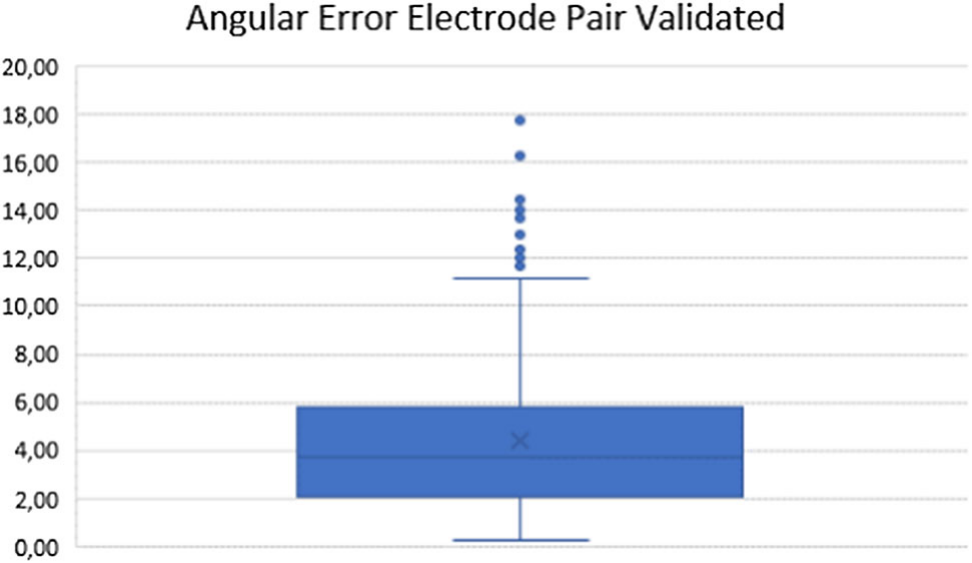
Angular error in degrees between two validated electrodes in a pair

Using the data from the validated electrode placements, the actual parallelism between each electrode pair was analysed. The median angle between electrode pairs was 3.80 (range 0.28–17.18) degrees (Table 3 and Fig. 5)

**Table 3:** Angular errors between validated electrode pairs

Median	3.80
Range	0.28–17.18

## Discussion

The use of a stereotactic CT-based navigation systems in placing single applicators for MWA and RFA has been demonstrated in previous publications. Wallach et al. used the CAScination system in a phantom and showed that stereotactic navigation with an aiming device (applicator guide) was superior to free hand navigation with the same system, with a lateral error 2.3 ± 1.3–2.8 ± 1.6 mm versus 4.2 ± 2.1 mm [8]. Engstrand et al. used the CAScination system for MWA and showed a lateral error of 4.0 ± 2.5 mm [9]. Beyer et al. compared CT-navigated stereotactic MWA using the CAS-ONE system with non-navigated conventional MWA using CT fluoroscopy. The accuracy was similar between the two methods, mean lateral error 2.8 mm SD 1.3. The main difference between the methods was the significantly lower radiation dose in the stereotactic group [10]. Lachenmeyer et al. also used the CAS-One system and showed a median lateral error of 3.2 (0.2–14.1) mm [11]. Bhattacharji et al. used a different system, IQQA-Guide, EDD Technology Inc, in a two-part study with both a phantom and in a clinical setting for both liver and lung lesions. The average needle placement accuracy in the liver phantom was 2.0 ± 0.7 mm – 2.8 ± 1.1 mm and 3.0 ± 1.4 mm in the lung. In the clinical setting, the accuracy was 4.6 ± 3.1 for all procedures [20]. Mbalisike et al. evaluated a robotic system (MAXIO, Perfint Healthcare, Indiana, USA) for applicator placement in MWA on patient that had been treated with trans-arterial chemoembolization (TACE) within 3 months prior to MWA. The robotic system had higher accuracy compared to CT-guided MWA with a mean applicator active point deviation (AAD) 5.3 ±1.8 mm versus 11.1 ±2.2 mm and the need for fewer applicator insertions and fewer readjustments [21]. Durand et al. used a different system with electromagnetic navigation system (IMATICS® CT navigation system) and compared it to conventional CT guided procedures. The navigation system improved accuracy, with distant error 4.1 mm versus 8.9 mm [22].

The placement of multiple electrodes at specific distances and in a parallel way that is required in IRE treatments is more demanding as discussed above. The accuracy of electrode placement in IRE treatment with the need for parallelism between the electrodes in each pair to achieve the recommended distance of 20 mm is one of the key points to a successful and safe treatment [6, 7].

This study is, so far, the largest regarding stereotactic navigation in IRE treatment. There are only a few other studies published. Beyer et al. compared CT-navigated stereotactic IRE electrode placement using the CAS-ONE system with CT fluoroscopy navigated electrodes with 10 patents in each group. The average deviation between IRE electrode and reference electrode was 2.2 mm (0.6–4.4mm) in the stereotactic group compared to 3.3 mm (0.6–6.2 mm) with a significantly lower radiation dose in the former group [14]. Beyer et al. have in another study compared free hand CT-fluoroscopy guided placement of IRE electrodes with a robotic system (Maxio, Perfint Healthcare, Florence, Oregon, USA). The difference in deviation with respect to the reference electrode was significantly better in the robotic group, 2.2 mm (0.0–4.0 mm) versus 3.1 mm (0.2–6.2 mm) [13].

In these studies, the accuracy is measured as lateral errors between the electrodes and the reference electrode. In this study, the angular error in comparison with the reference electrode is measured with a mean of 1.88 ° (SD 2.11°). In addition to this, the lateral and angular errors between planned and placed electrode have been analysed with a mean error of 4.05 mm (SD 2.60 mm) and 3.62° (SD 2.40°). These numbers describe the electrode placement more accurately and validate the efficacy of the stereotactic navigation system.

This study shows that the accuracy in placing the electrodes where the interventionist has planned to place them is well within the error margins for completing an adequate tumour ablation. The clinical follow-up shows no electrode placement-related complications. A few electrodes were not accurate enough and had to be re-positioned before the treatment.

The analysis of the parallelism between each electrode pair shows that the stereotactic navigation is an efficient tool in placing multiple electrodes accurately.

The use of CT-based navigation system makes it easy to analyse electrode placement after the treatment since all images are saved in the same coordinate space system. It is also an advantage that the planning performed on the screen can be evaluated by multiple clinicians before the electrode is placed. This is also an advantage in the process of learning the methodology.

One of the disadvantages of the method is the need for almost perfect muscle relaxation since the first contrast enhanced CT scan is the base for the treatment and all the electrode validation CT images are fused with this and if the target organ has moved, the accuracy of the electrode placement is very difficult to perform. With a team of dedicated anaesthesiologists and anaesthesiology nurses in the ablation team, this is a minor issue.

When an ablation centre starts using the system, there is a learning curve in optimizing the workflow. The use of HFJW, attachment of the skin-markers, the first CT scan and the planning are all moments that can be optimized, and the total procedure times will decrease with experience.

The single centre set-up of this study is a limitation and will affect the generalizability of the results.

## Conclusion

The use of a stereotactic CT-based navigation system is a user friendly, accurate and safe way of placing multiple electrodes.
